# An optimized protocol to detect high‐throughput DNA methylation from custom targeted sequences on 96 samples simultaneously

**DOI:** 10.1002/2211-5463.70093

**Published:** 2025-08-13

**Authors:** Nathalie Iannuccelli, Sophie Valière, Julien Sarry, Cécile Donnadieu, Julie Demars

**Affiliations:** ^1^ GenPhySE, Université de Toulouse, INRAE, ENVT Castanet‐Tolosan France; ^2^ INRAE, GeT‐PlaGe, Genotoul Castanet‐Tolosan France

**Keywords:** biotechnology, custom capture, DNA methylation, enzymatic conversion, hybridization, molecular biology

## Abstract

Genome methylation represents an important source of regulation of gene expression. To date, custom molecular tools for studying targeted regions of the genome are restricted to several megabases. We developed a protocol to epigenotype differentially methylated CpGs in specific regions of the genome. The protocol describes a targeted methylation library preparation upstream short read sequencing with an Illumina instrument. The protocol includes the New England Biolabs Next Enzymatic Methyl‐seq Library Preparation workflow combined with the Twist Bioscience Targeted Methylation Sequencing workflow. The protocol is divided into eight steps: fragmentation, library preparation, enzymatic conversion, indexing, pooling, hybridization, capture, and amplification. Main advantages are (a) a lower amount of DNA (100 and 50 ng) than other technologies, (b) the limitation of DNA degradation using enzymatic conversion instead of chemical bisulfite, (c) the pooling of samples into 8‐plex reducing handling time, and (d) the significant reduction of the panel quantity divided by 20 for saving experimental costs. This protocol was carried out on 96 samples simultaneously in a standard molecular biology laboratory, and the multiplexing can be run up to 384 samples for methylation experiments. We developed a high‐throughput epigenotyping method as an alternative of methylation arrays. This approach can be adapted to any interesting regions using a custom panel for agronomic species and model organisms.

## Graphical abstract

Workflow of a high‐throughput technology for epigenotyping of differentially methylated CpGs in specific regions of the genome. The protocol works with small amounts of DNA extracted from blood or semen. The protocol consists of both enzymatic conversion of unmethylated cytosines and capture by hybridization with a custom panel. The protocol is adaptable to any species, tissues, or regions.

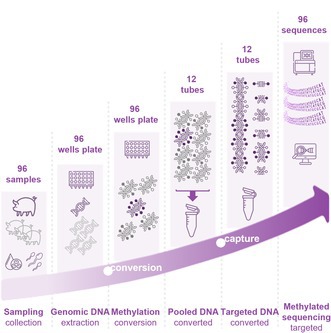

**Abbreviations**
5mC: 5 methyl cytosine
APOBEC: apolipoprotein B mRNA editing enzyme, catalytic polypeptide‐like
BSA: Bovin Serum Albumin
CpG: cytosine phosphate guanine
DTT: dithiothreitol
EMBL‐EBI: European Molecular Biology Laboratory‐European Bioinformatics Institute
ENA: European Nucleotide Archive
epiWAS: epigenomic‐wide association study
qPCR: quantitative polymerase chain reaction
TE: tris EDTA
TET2: tet methylcytosine dioxygenase 2

Studies of epigenetic modifications such as DNA methylation are essential for understanding the regulation of gene expression. Many approaches are developed to identify and quantify DNA methylation across the whole genome or targeted regions. The methylation is detected thanks to the conversion of unmethylated 5‐cytosines to uracil either by chemical bisulfite or enzymatic reaction. Different available technologies, based on microarray or sequencing methods, are benchmarked on numerous recent scientific publications [1, 2, 3, 4, 5, 6]. As an example, Tanić *et al*. [2] benchmarked five commercially available targeted‐bisulfite sequencing platforms that were all performed in human samples. All these experiments relied on the amount of input DNA ranging from 100 ng to 1 μg. In addition, all sequencing methods relied on 96 multiplexing samples. Although existing technologies developed in humans allow simultaneous targeting of large, numerous, and specific CpG‐rich regions across the genome, the size of targeted regions remains homogeneous since they are based on specific genomic features. In addition, for custom capture‐based technology, the overall size of panels is restricted to 10 Mb and less [7]. Most technologies are based on bisulfite conversion known to damage DNA molecules. Capture is usually performed before the conversion step, which reduces the performance of the capture to the targeted regions. Indeed, available and standard protocols use genomic probes to capture genomic regions. The methylation bisulfite conversion step is performed on the captured fraction, leading to the degradation of DNA and a lesser representation of targeted sequences [8].

To overcome these limitations, we optimized a protocol to target specific CpG highly rich regions using enzymatic conversion followed by a custom capture. We adapted two existing protocols: New England Biolabs Next Enzymatic Methyl‐seq Library Preparation Protocol and Twist Bioscience Targeted Methylation Sequencing Protocol. The workflow is divided into eight steps: fragmentation, library preparation, enzymatic conversion, indexing, pooling, hybridization, capture, and amplification.

We described a step‐by‐step process from genomic DNA fragmentation to sequencing.

Recently, we published a scientific article comparing our technology with the benchmark technology [7]. Here, we upgraded the eight samples protocol to a high‐throughput 96 samples simultaneous protocol.

DNA was mechanically fragmented and ends were repaired. After ligation of methylated adapters, fragments were purified. Then, the 5mC were oxidized by the TET2 enzyme and an additional APOBEC treatment deaminated the unmethylated cytosines to uracils. After purification, the converted library was indexed by PCR amplification. A quality control was done, and eight libraries were pooled in equimolar quantity. Then, hybridization with a custom double‐stranded DNA panel was performed to target specific regions. Fragments of interest were captured with streptavidin beads. After PCR amplification, each pool of eight libraries was quantified by qPCR before sequencing. The protocol was carried out on 12 pools of eight libraries; consequently, 96 samples were processed simultaneously.

The protocol described works with small amounts of input DNA extracted from different tissues such as mammalian blood or semen. The protocol works with a large custom panel covering highly CpG‐rich regions, and it could be adapted to any region, as it uses custom probes. However, we recommend adapting: (a) the quantity of probes according to the size of the panel, (b) the number of PCR cycles according to the size of the panel, and (c) the hybridization washing temperature to adjust the panel specificity. This approach could be used for methylation studies such as epiWAS of any organisms that have a reference genome available and also in humans as a high‐density diagnostic tool.

## Materials

### Reagents

- Twist Targeted Methylation Sequencing Workflow (Twist Bioscience, 103496, South San Francisco, CA, USA)
- Twist Methyl Custom panel (Twist Bioscience, 0.01 fmol per probe per 4 μL)
- Qubit DS DNA Broad Range Assay kit (ThermoFisher Scientific, Q32850, Waltham, MA, USA)
- Qubit DS DNA High Sensitivity Assay kit (ThermoFisher Scientific, Q32851)
- Fragment Analyzer High Sensitivity NGS kit (Agilent Technologies, DNF474, Santa Clara, CA, USA)

### Equipment

- Pixul ultrasonicator (Active Motif)
- Thermal Cycler 96 well, temperature‐adjustable heated lid
- Speed Vacuum concentrator
- Qubit fluorometer (Thermo Fisher Scientific)
- Fragment Analyzer (Advanced Analytical, Heidelberg, Germany)

## Methods

### Ethics approval and consent to participate

All procedures were conducted in accordance with the French legislation on animal experimentation and ethics. Julie Demars was authorized by the French ministry of Agriculture to conduct experiments on living animals at the INRAE facilities in Toulouse, France (approval number ENVT‐FC‐DE‐074/2017). The experiment authorization number of the experimental farm GenESI (https://doi.org/10.15454/1.5572415481185847E12) is 11789‐2017101117033530 v8. The study included 96 pigs born on the INRAE experimental farm in accordance with the French and European legislation on animal welfare. All the procedures and guidelines for animal care were approved by the local ethical committee in animal experimentation (Poitou–Charentes) and the French ministry of Higher Education and Scientific Research (authorization n° 11789‐2017101117033530).

**NEBNext Enzymatic Methyl‐Seq Protocol Standard Insert Library (370–420 bp)** (DOC‐001224 REV 4.0)

**Fig. 1:**
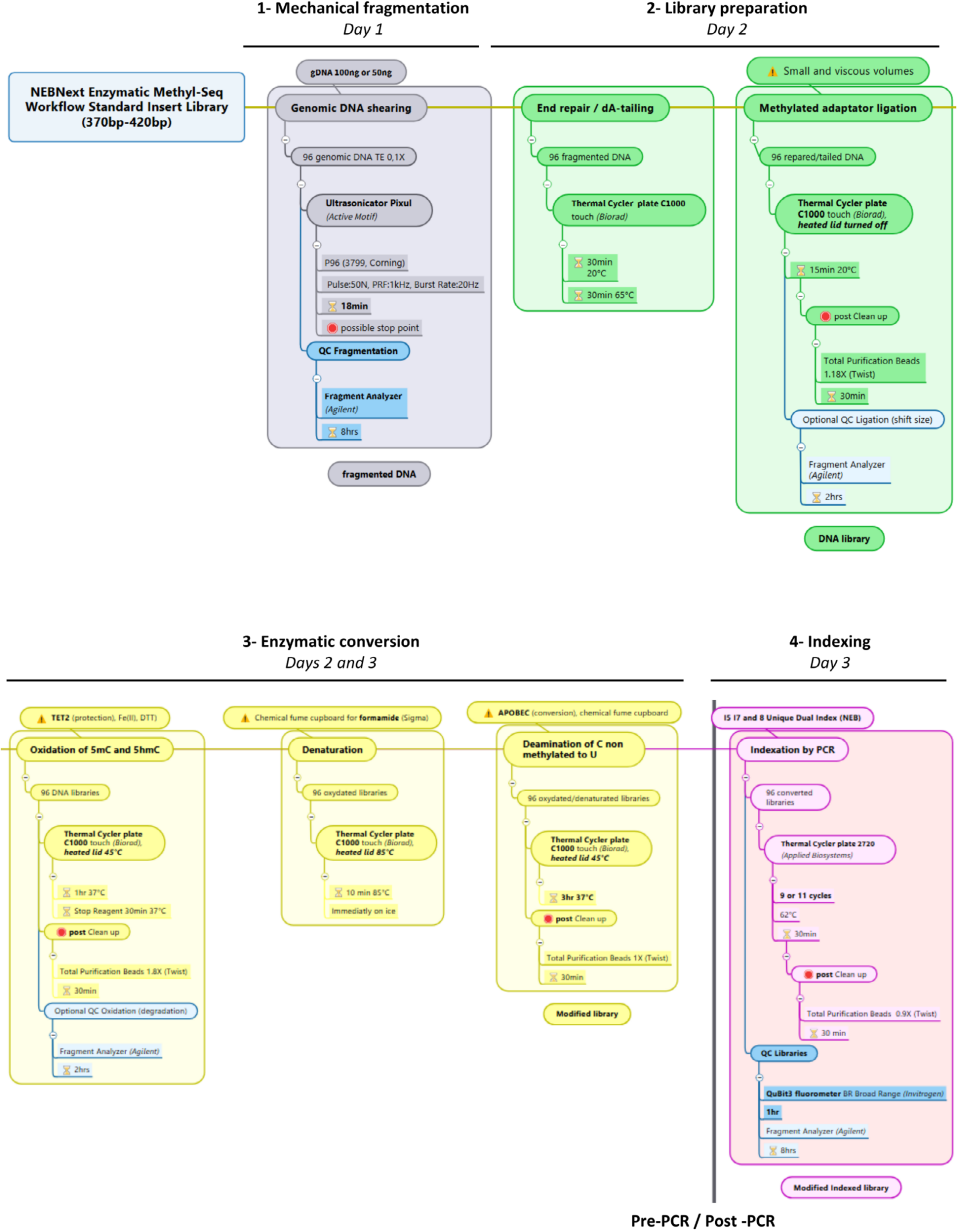
A schematic overview of the first part of the whole workflow (steps 1 to 4).


The whole workflow of the first part (steps 1 to 4) of the protocol is presented in Fig. 1.

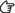
 All reagents thaw on ice unless specific recommendations, then pulse‐vortex for 2 s to mix, and followed by pulse‐spin

1. Mechanical fragmentation

1.1. Genomic DNA preparation

▢ Dilute genomic DNA (100 ng* or 200 ng**, fluorometric assay) to 100 μL with 0.1× TE buffer directly in a well plate recommended for Pixul fragmentation


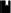
 *100 ng per semen extraction and **200 ng per blood extraction

● Possible stop point but avoid the DNA freeze–thaw circles

1.2. DNA shearing

▢ Fragment the DNA using Pixul ultrasonication parameters

Pulse: 50 N

PRF: 1 kHz

Burst rate: 20 Hz

Duration: 18 min

● Possible stop point post‐fragmentation but avoid the DNA freeze–thaw circles

1.3. QC fragmentation

**Fig. 2:**
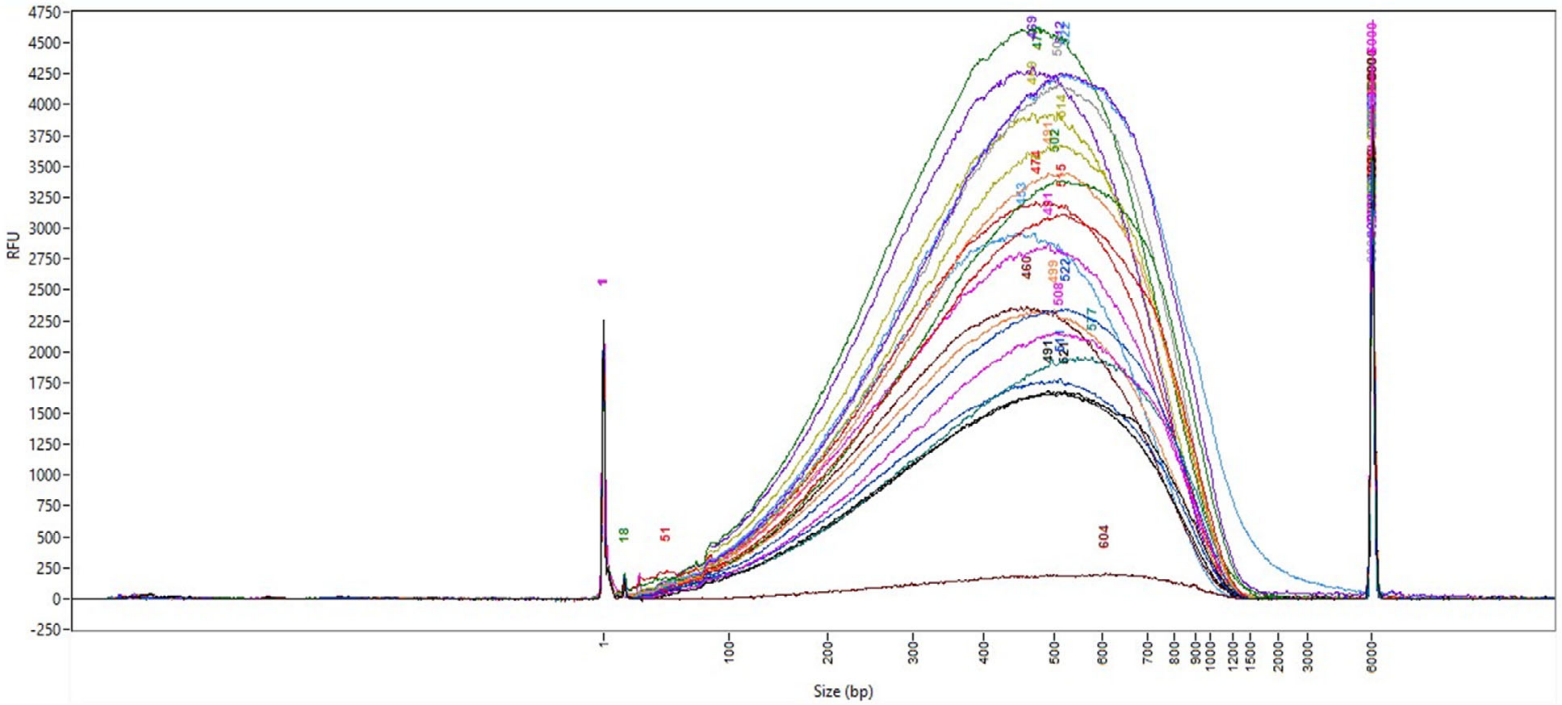
Size distribution of a subset of 96 samples (*n* = 16) fragmented DNA with Pixul ultrasonicator (Active Motif) on a Fragment Analyzer with High Sensitivity DNA kit (Agilent Technologies). The curves represent the RFU (Relative Fluorescence Units) values of signal intensity measurements as a function of fragment size. Each curve corresponds to one sample.


Validation is performed on a Fragment Analyzer with High Sensitivity NGS Fragment Analysis kit according to manufacturer's recommendations (Fig. 2).

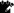
 Average size distribution should be approximately 450–550 bp

2 Library preparation

2.1. End repair/dA‐tailing

▢ Set the heated lid of the thermocycler to 75 °C

▢ Transfer 50 μL of the sheared DNA (or sheared DNA Controls) in a well plate recommended for the thermocycler

▢ On ice, add the following components to sheared DNA:

▢ NEBNext Ultra II End prep Reaction Buffer: 7 μL

▢ NEBNext Ultra II End prep Enzyme Mix: 3 μL

Total volume: 60 μL

▢ Gently pipette the entire volume up and down at least 10 times to mix thoroughly

▢ Perform a quick spin to collect all liquid from the sides of the well plate


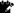
 The presence of a small amount of bubbles will not interfere with performance

▢ Place the plate in a thermocycler and run the following program:

**Table:** 

Temperature	Time	Lid 75 °C
20 °C	30 min	
65 °C	30 min	
4 °C	Hold	

2.2. Methylated adaptor ligation

▢ On ice, add the following components to repaired DNA:

▢ NEBNext^®^ EM‐seq™ Adaptor: 2.5 μL

Total volume: 62.5 μL

▢ Gently pipette the entire volume up and down at least 10 times to **mix thoroughly**

▢ Perform a quick spin to collect all liquid from the sides of the well plate

▢ On ice, add the following components to repaired DNA:

▢ NEBNext^®^ Ligation Enhancer: 1 μL

▢ NEBNext^®^ Ultra™ II Ligation Master Mix: 30 μL

Total volume: 93.5 μL


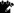
 The Ligation Master **Mix is viscous. Pipette very slowly**, watch reagent slowly creep down pipette tip


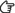
 For multiple reactions, a master mix of the above reaction components can be prepared before addition to the mix sample/adaptor

▢ Gently pipette the entire volume up and down at least 10 times to **mix thoroughly**

▢ Perform a quick spin to collect all liquid from the sides of the well plate


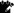
 The presence of small amount of bubbles will not interfere with performance

▢ Incubate at 20 °C for 15 min in a thermocycler **with the heated lid turned off**

● Safe stopping point: Sample can be stored overnight at −20 °C

2.3. Clean‐up of adaptor ligated DNA

▢ Vortex Total Purification Beads

▢ Equilibrate Total Purification Beads to room temperature (30 min)

▢ Vortex Total Purification Beads until homogenization

▢ Add 110 μL (1.18×) of homogenized beads to each sample

▢ Mix well by vortexing

▢ Incubate 5 min at room temperature

▢ Place the plate against an appropriate magnetic stand for 5 min to separate the beads from the supernatant

▢ Carefully remove and discard supernatant


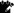
 Do not discard beads pellet that contain DNA targets

▢ Add 200 μL of **freshly** prepared 80% ethanol to gently wash the beads pellet


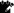
 Do not disturb the bead pellet

▢ Incubate 1 min

▢ Carefully remove and discard ethanol


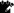
 Do not discard beads that contain DNA targets

▢ ▢ Repeat the wash 2 more times with **freshly** prepared 80% ethanol

▢ Place the plate against an appropriate magnetic stand


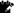
 Be sure to remove all visible liquid using a 10 μL pipette tip

▢ Air‐dry the beads pellet for 2 min


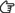
 Do not over‐dry the beads pellet to not reduce DNA recovery. The beads must still remain dark brown and glossy looking; the beads must not turn lighter and start to crack

▢ Remove the plate from the magnetic stand

▢ Add 30 μL of Elution Buffer to the beads pellet

▢ Mix well by pipetting up and down 10 times

▢ Incubate 2 min at room temperature

▢ Place the plate against an appropriate magnetic stand for 3 min to separate the beads from the supernatant

▢ Transfer 28 μL of the clear supernatant containing the adaptor ligated DNA to a new well plate


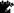
 Make sure not to disturb the beads pellet

● Safe stopping point: Sample can be stored overnight at −20 °C.

3 Enzymatic conversion

3.1. Oxidation of 5mC and 5hmC

▢ Add 400 μL of TET2 Reaction Buffer to TET2 Reaction Buffer Supplement powder

▢ Mix well by vortexing


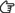
 Reconstituted TET2 Reaction Buffer should be stored at −20 °C and discarded after 4 months.

▢ On ice, add the following components to the 28 μL adaptor ligated DNA:

**Table:** 

**Reconstituted** TET2 Reaction Buffer	10 μL
Oxidation Supplement	1 μL
DTT	1 μL
Oxidation Enhancer	1 μL
TET2	4 μL

Volume total: 45 μL


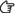
 For multiple reactions, a master mix of the above reaction components can be prepared before addition to the sample

▢ Mix thoroughly by vortexing

▢ Centrifuge briefly

▢ Dilute the 500 nm Fe (II) solution by adding 1 to 1249 μL of nuclease‐free water


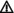
 **Use the diluted Fe (II) solution immediately; discard after use**

▢ Add 5 μL of **diluted** Fe (II) solution to the well plate to initiate the oxidation reaction

Volume total: 50 μL

▢ Mix well by vortexing

▢ Centrifuge briefly

▢ Incubate at 37 °C for 1 h in a thermocycler lid set to 45 °C

▢ Transfer the sample on ice

▢ Add 1 μL of Stop Reagent

▢ Mix well by vortexing

▢ Centrifuge briefly

▢ Incubate at 37 °C for 30 min, lid set to 45 °C, then at 4 °C in a thermocycler.

● Possible stopping point: Sample can be stored overnight at either 4 °C in the thermocycler or at −20 °C in the freeze.

3.2. Clean‐up of oxidated DNA (see 2.3)

▢ Adding 90 μL (1.8×) of homogenized beads to each sample

▢ Adding 18 μL of Elution Buffer to the beads pellet to transfer 16 μL of the clear supernatant containing the adaptor ligated DNA to a new well plate

● Safe stopping point: Sample can be stored overnight at −20 °C

3.3. **Denaturation** (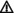 under chemical fume cupboard)

▢ Pre‐heat a thermocycler to 85 °C

▢ Add 4 μL formamide to the 16 μL of oxidized DNA

▢ Mix well by vortexing

▢ Centrifuge briefly

▢ Incubate at 85 °C for 10 min in the ***preheated*** thermocycler lid set to 85 °C

▢ **Immediately** place on ice

▢ Centrifuge briefly

3.4. **Deamination of C to U** (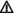 under chemical fume cupboard)

▢ On ice, add **immediately** the following components to the 20 μL denatured DNA:

**Table:** 

Nuclease water	68 μL
APOBEC Reaction Buffer	10 μL
BSA	1 μL
APOBEC	1 μL

Volume total: 100 μL


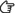
 For multiple reactions, a master mix of the above reaction components can be prepared before addition to the denatured DNA

▢ Mix well by vortexing

▢ Centrifuge briefly

▢ Incubate at 37 °C for 3 h, lid set to 45 °C, then at 4 °C in a thermocycler

● Possible stopping point: Sample can be stored overnight at either 4 °C in the thermocycler or at −20 °C in the freezer

3.5. **Clean‐up of deaminated DNA** (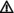 under chemical fume cupboard) (see 2.3)

▢ Adding 100 μL (1×) of homogenized beads to each sample

▢ Adding 22 μL of Elution Buffer to the beads pellet to transfer 20 μL of the clear supernatant containing the deaminated DNA to a new well plate


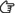
 **The beads behave differently during APOBEC clean‐u; do not over‐dry the beads as they become very difficult to resuspend**

● Safe stopping point: Sample can be stored overnight at −20 °C

4 Indexing

4.1. Indexation by PCR

▢ On ice, add the following components to the 20 μL deaminated DNA:

**Table:** 

EM‐Seq Index Primer tube (10 μm)	5 μL
NEBNext^®^ Q5U^®^ Master Mix	25 μL

Total volume: 50 μL

▢ Mix well by vortexing

▢ Centrifuge briefly

▢ Place the plate in a thermocycler and run the following program:

**Table:** 

Cycles step	Temperature	Time	Cycles^2^
Initial denaturation	98 °C	30 s	1
Denaturation	98 °C	10 s	
Annealing	62 °C	30 s	9* or 11**
Extension	65 °C	60 s	
Final extension	65 °C	5 min	1
Hold	4 °C		


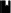
 *9 cycles for 100 ng input and **11 cycles for 50 ng input

● Possible stopping point: Sample can be stored overnight at either 4 °C in the thermocycler or at −20 °C in the freezer

4.2. Clean‐up indexed library (see 3.2)

▢ Adding 45 μL (0.9×) of homogenized beads to each sample

▢ Adding 22 μL of Elution Buffer to the beads pellet to transfer 20 μL of the clear supernatant containing the indexed DNA library to a new well plate

● Safe stopping point: Sample can be stored overnight at −20 °C

4.3. QC libraries

4.3.1. ***Quantification*** is performed on a Qubit 3.0 fluorometer with DS DNA Broad Range Quantitation Assay kit according to manufacturer's recommendations.

Average concentration of each library should be approximately 30–50 ng·μL^−1^

**Fig. 3:**
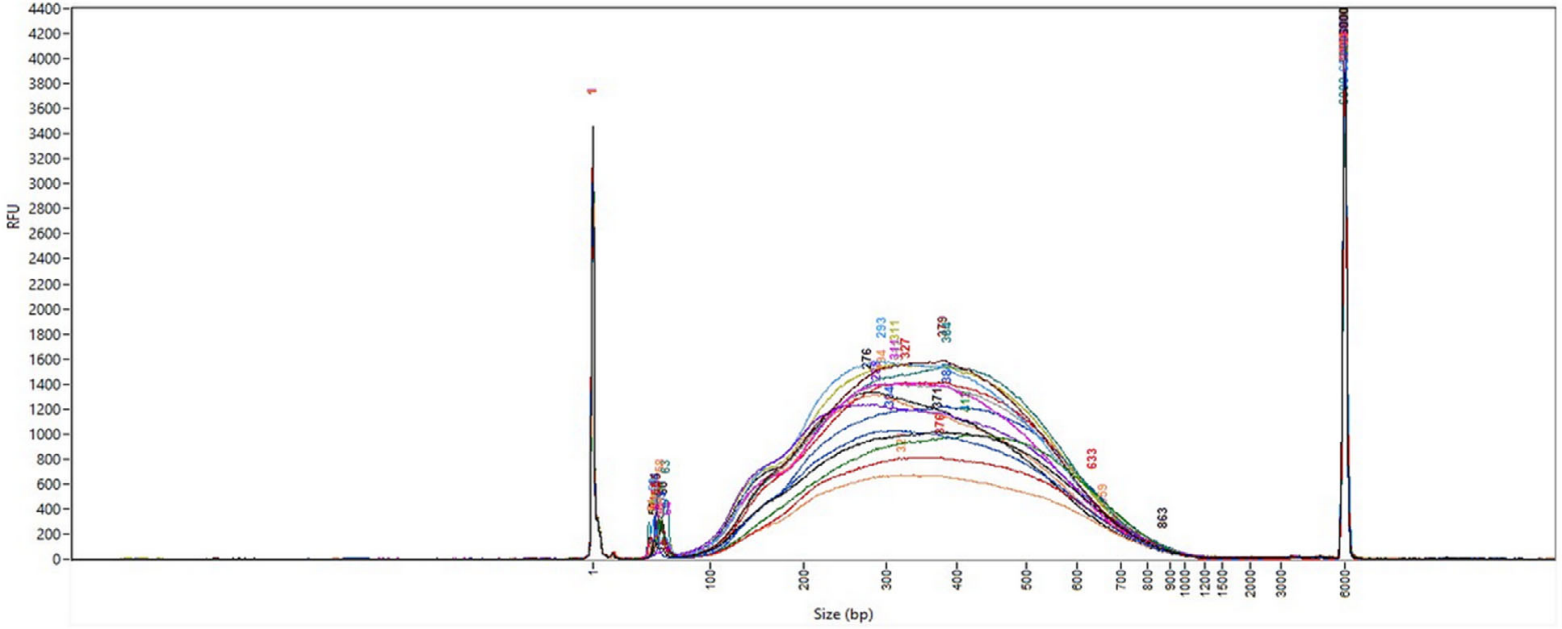
Size distribution of a subset of 96 samples (*n* = 16) EMSeq NEB libraries on a Fragment Analyzer with High Sensitivity DNA kit (Agilent Technologies). The curves represent the RFU (Relative Fluorescence Units) values of signal intensity measurements as a function of fragment size. Each curve corresponds to one sample.


4.3.2. ***Validation*** is performed on a Fragment Analyzer with High Sensitivity NGS Fragment Analysis kit according to manufacturer's recommendations (Fig. 3)

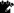
 Average size distribution should be approximately 350–450 bp

**Twist Bioscience Targeted Methylation Sequencing Protocol** (DOC‐001222 REV 4.0)

**Fig. 4:**
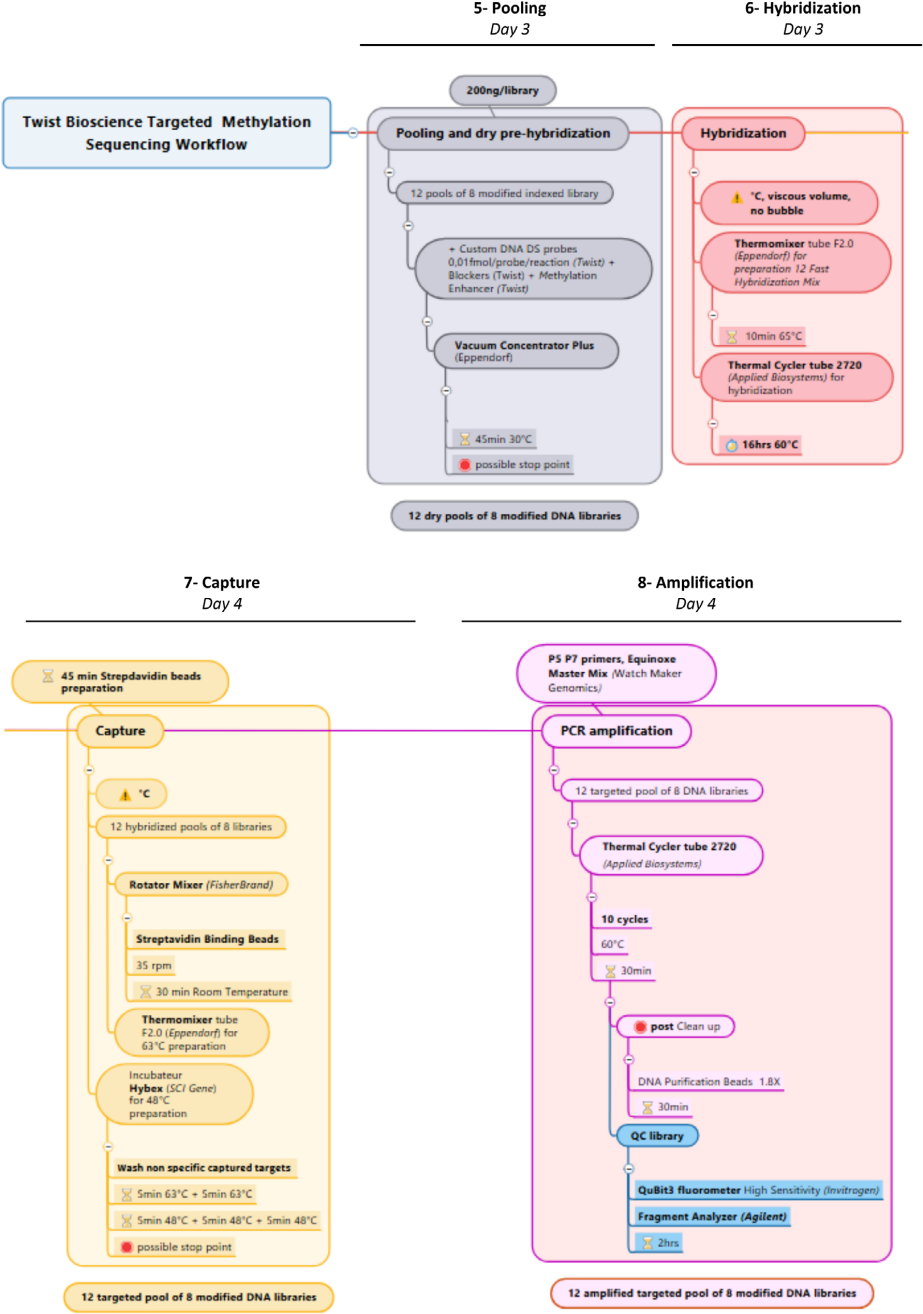
A schematic overview of the second part of the whole workflow (steps 5 to 8).


The whole workflow of the second part (steps 5 to 8) of the protocol is presented in Fig. 4.

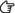
 All reagents thaw on ice unless otherwise specified, then pulse‐vortex 2 s to mix and pulse‐spin

5 **Pooling** of 8 samples

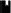
 Total DNA amount per pool depends on the number of libraries in the pool


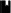
 Total DNA amount per pool of 8 samples should be 1500 ng


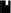
 The amount of each library per pool will be the same***

▢ Transfer the calculated volumes from each indexed library **in a PCR 0.2 mL tube appropriate for the hybridization reaction later**

▢ Add the following reagents to the pool of 8 indexed libraries:

**Table:** 

Twist Bioscience **Custom** Methylation Panel^Custom5.a^	4 μL
Universal Blocker	8 μL
Blocker Solution	5 μL
Methylation Enhancer	2 μL^Custom5.b^

▢ Mix by flicking the 0.2 mL tube

▢ Perform a quick spin


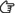
 Ensure there are minimal bubbles present

▢ Dry the pre‐hybridization solution using a vacuum concentrator into 0.2 mL appropriate support at low heat (30 °C) about 30 min

● Safe stopping point: Dry pre‐hybridization solution can be stored overnight at −20 °C

6 Hybridization

▢ Pre‐heat a Thermal Cycler at 95 °C

▢ Pre‐heat a Thermo mixer at 65 °C

▢ Thaw Fast Hybridization Mix at room temperature

▢ Thaw Hybridization Enhancer at room temperature

▢ Heat 22 μL of Fast Hybridization Mix at 65 °C for 10 min (for each pool)


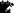
 Do not allow the Fast Hybridization Mix to cool to room temperature

▢ Resuspend carefully the dried pre‐hybridization material with 20 μL preheated Fast Hybridization Mix


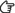
 Fast Hybridization Mix is **viscous**, **dispense slowly** to ensure accuracy


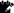
 The presence of small particles in the custom methylation panel will not interfere with performance

▢ Perform a spin


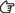
 Ensure there are no bubbles present to limit off target

▢ Add 30 μL of Hybridization Enhancer to the top of the pre‐hybridization solution


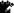
 Hybridization enhancer is mineral oil to prevent evaporation


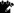
 Hybridization enhancer settles on top of the hybridization reaction does not affect the final captured product

▢ Place the 0.2 mL tube of hybridization solution in the **preheated** 95 °C thermal cycler and run the following program:

**Table:** 

Temperature	Time
95 °C	5 min
60 °C^Custom6.a^	16 h^Custom6.b^


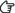
 Make sure the 0.2 mL tube is sealed tightly to prevent evaporation during the incubation

7 Capture

7.1. Streptavidin beads preparation

▢ Vortex Streptavidin Binding beads

▢ Equilibrate Streptavidin Binding beads to room temperature (at least 30 min)

▢ Equilibrate Fast Binding Buffer to room temperature

▢ Vortex Streptavidin Binding Beads until mixed well

▢ **For each capture reaction**, add 100 μL Streptavidin Binding Beads to a clean 1.5 mL LoBind tube (1 tube per hybridization)

▢ Add 200 μL Fast Binding Buffer

▢ Mix by pipetting until mixed well

▢ Place on a compatible magnetic stand

▢ Incubate 1 min at room temperature

▢ Carefully remove and discard supernatant


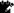
 Do not disturb the bead pellet

▢ Remove from the magnetic stand

▢ Add 200 μL Fast Binding Buffer

▢ Mix by vortexing until mixed well

▢ Place on a compatible magnetic stand

▢ Incubate 1 min at room temperature

▢ Carefully remove and discard supernatant


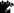
 Do not disturb the bead pellet

▢ Remove from the magnetic stand

▢ Add 200 μL Fast Binding Buffer

▢ Mix by vortexing until mixed well

▢ Place on a compatible magnetic stand

▢ Incubate 1 min at room temperature

▢ Carefully remove and discard supernatant


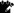
 Do not disturb the bead pellet

▢ Remove from the magnetic stand.

▢ Add 200 μL Fast Binding Buffer.

▢ Resuspend washed beads by vortexing until homogenized.

7.2. Streptavidin binding beads


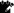
 After hybridization is complete, **do not remove the tube from thethermocycler**

▢ Transfer **quickly and directly** the full volume of one hybridization into a 1.5 mL tube of **washed** streptavidin beads


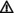
 **Rapid transfer directly from the thermal cycler is a critical step for minimizing off‐target binding**


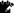
 Do not disturb the bead pellet

▢ Mix by pipetting and flicking


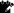
 Do not vortex, aggressive mixing is not required

▢ Mix on a rotator mixer for 30 min at room temperature at a sufficient speed to keep the solution mixed (35 r.p.m.)

7.3. Wash no specific captured targets

▢ Heat at 48 °C^Custom7.3^ Fast Wash Buffer 1 and Wash Buffer 2 in Heat Block Hybex

▢ Pre‐heat 420 μL Fast Wash Buffer 1 to 63 °C in a dry bath incubator (for each pool, 1 pool per 1.5 mL tube)

▢ Pre‐heat 610 μL Wash Buffer 2 to 48 °C in Heat Block Hybex block 1.5 mL (for each pool, 1 pool per 1.5 mL tube)

▢ Remove the 1.5 mL tube containing the hybridization reaction with streptavidin binding beads from the rotator mixer

▢ Perform a quick spin to ensure that the whole solution is at the bottom of the 1.5 mL tube

▢ Place on a compatible magnetic stand

▢ Incubate 1 min at room temperature

▢ Carefully remove and discard supernatant, including hybridization enhancer


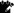
 Do not discard beads pellet that contain DNA targets


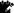
 Trace amount of hybridization enhancer may be visible after supernatant removal, but it will not affect the final capture product

▢ Remove the 1.5 mL tube from the magnetic stand

▢ Add 200 μL **preheated 63 °C Fast Wash Buffer 1**

▢ Mix by pipetting

▢ Perform a quick spin

▢ Incubate 5 min at 63 °C in the preheated Dry Bath Incubator

▢ Place the 1.5 mL tube on a compatible magnetic stand

▢ Incubate 1 min at room temperature

▢ Carefully remove and discard clear supernatant


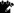
 Do not discard beads pellet that contain DNA targets

▢ Remove the 1.5 mL tube from the magnetic stand

▢ Add 200 μL **preheated 63 °C Fast Wash Buffer 1**

▢ Mix by pipetting

▢ Perform a quick spin

▢ Incubate 5 min at 63 °C in the preheated Dry Bath Incubator

▢ Perform a quick spin to ensure all solution is at the bottom of the 1.5 mL tube

▢ Transfer the entire volume to a new 1.5 mL tube


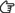
 **This step reduces background resulting from non‐specific binding to the surface of the 1.5 mL tube**

▢ Place the 1.5 mL tube on a compatible magnetic stand

▢ Incubate 1 min at room temperature

▢ Carefully remove and discard clear supernatant


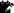
 Do not discard beads pellet that contain DNA targets

▢ Remove the 1.5 mL tube from the magnetic stand

▢ Add 200 μL **preheated 48 °C Wash Buffer 2**

▢ Mix by pipetting

▢ Perform a quick spin

▢ Incubate 5 min at 48 °C in preheated Heat Block Hybex

▢ Place the 1.5 mL tube on a compatible magnetic stand

▢ Incubate 1 min at room temperature

▢ Carefully remove and discard clear supernatant


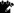
 Do not discard beads pellet that contain DNA targets

▢ ▢ Repeat the wash 2 more times with **48 °C Wash Buffer 2**

▢ Remove all traces of supernatant using a 10 μL pipette tip


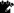
 Before pipetting, the beads pellet may be briefly spun to collect supernatant at the bottom of the 1.5 mL tube and returned to the magnetic stand


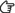
 **Proceed immediately to the next step, do not allow the beads to dry**

▢ Remove the 1.5 mL tube from the magnetic stand

▢ Add 45 μL molecular biology grade water

▢ Mix by pipetting until homogenization

▢ Place this solution of streptavidin binding beads **slurry** on ice

● Safe stopping point: slurry can be stored at −20 °C for 1 month

8 Amplification

8.1. PCR amplification

▢ Mix by pipetting streptavidin binding beads slurry

▢ Transfer 22.5 μL streptavidin binding beads slurry to a new 0.2 mL tube appropriate for the thermal cycler


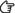
 Store the remaining 22.5 μL streptavidin binding beads slurry at −20 °C for future use.

▢ On ice, add the following reagents to the 0.2 mL tube containing streptavidin binding beads slurry:

**Table:** 

P5P7 Primers Mix (10 μm)	2.5 μL
Equinox Library Amplification Mix (2×)	25 μL

Total volume: 50 μL

▢ Mix gently by pipetting

▢ Perform a quick spin to collect all liquid from the sides of the 0.2 mL tube

▢ Place the 0.2 mL tube in a thermocycler and run the following program:

**Table:** 

Cycles step	Temperature	Time	Cycles
Initial denaturation	98 °C	45 s	1
Denaturation	98 °C	15 s	
Annealing	60 °C	30 s	10^Custom8.1^
Extension	72 °C	30 s	
Final extension	72 °C	1 min	1
Hold	4 °C		


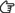
 **Proceed immediately to the next step**

8.2. Clean‐up of amplified library

▢ Adding 90 μL (1.8×) of homogenized beads to each sample

▢ Adding 32 μL of Elution Buffer (white) to the beads pellet to transfer 30 μL of the clear supernatant containing the target's DNA to a new PCR 0.2 mL tube

● Safe stopping point: Sample can be stored overnight at −20 °C

8.3. QC library

8.3.1. ***Quantification*** is performed on a Qubit 3.0 fluorometer with DS DNA High Sensitivity Quantitation Assay kit according to manufacturer's recommendations

Average concentration of the library pools should be approximately 5–30 ng·μL^−1^.

**Fig. 5:**
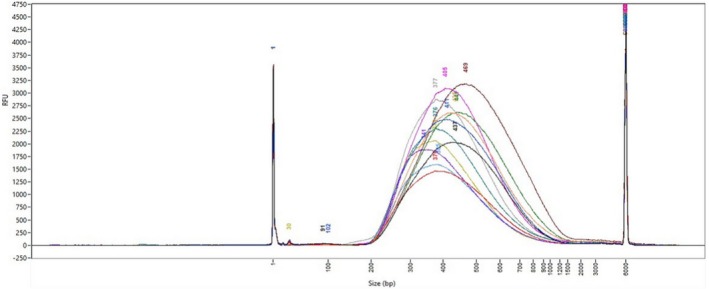
Size distribution of the 96 samples pooled in 12 Twist Bioscience targeted libraries on a Fragment Analyzer with High Sensitivity DNA kit (Agilent Technologies). The curves represent the RFU (Relative Fluorescence Units) values of signal intensity measurements as a function of fragment size. Each curve corresponds to one pool of eight samples.


8.3.2. ***Validation*** is performed on a Fragment Analyzer with High Sensitivity NGS Fragment Analysis kit according to manufacturer's recommendations (Fig. 5)

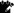
 Average size should be approximately 350–450 bp

## Tips & tricks/troubleshooting

1. Biological sample

Genomic DNA was extracted either from blood using an in‐house procedure of proteinase K digestion and ethanol precipitation with salts, or from semen using an in‐house procedure of phenol–chloroform extraction. The manufacturer recommends 200 ng of genomic DNA input. We have successfully reduced this to 100 ng DNA input for blood DNA and 50 ng input for semen DNA. This improvement is interesting for processing precious samples available in small quantities.

2 Product

The formamide should be aliquoted and stored at −20 °C to avoid the formation of formic acid that degrades DNA.

3 Protocol

The complete protocol takes 4 working days, but there are several steps at which the procedure can be paused (stopping points), allowing some flexibility in the workflow. Depending on the number of Quality Controls performed (2 or more) the workflow time can be reduced. The protocol can be carried out by 2 people in parallel to avoid magnetic beads drying out during the various washes, preventing loss of yield. A previous version of the protocol was carried out on eight samples [7]. Here, the development of high‐throughput requires (a) working on plates instead of tubes, (b) using the Pixul instrument for quick and homogeneous mechanical fragmentation in plates instead of the Covaris instrument with manual and individual sonication, and (c) minimizing the time of DNA beads purification, which is particularly critical at the enzymatic conversion step. In addition, the main deviations from the standard Twist protocol totally depend on the design of our custom panel, including a large size of over 20 Mb, a heterogeneity of targeted region sizes ranging from 400 bp to 2 Mb, and CpG highly rich regions. Recommendations to be taken have been directly written into the procedure, but the most important recommendations, including the use of a custom panel, are listed below in each section.

- Fragmentation

We used a Pixul instrument to process 96 samples simultaneously in a 96‐well plate. This high‐speed instrument is less expensive than the Covaris reference instrument and can be used to prepare any NGS libraries.

We recommend using the NEBNext Enzymatic Methyl‐Seq Protocol Standard Insert Library (370–420 bp), not the Large Insert Library (470–520 bp). Then perform 2 × 100 or 2 × 150 bp sequencing, depending on the fragmentation time and sequencing equipment available.

- Library preparation

No supplementary recommendations.

- Enzymatic conversion

No supplementary recommendations.

- Indexing

As multiple 384 indexes are now provided and available (Twist Biosciences, 107518, 107523 and 107524), 384 sample multiplexing sequencing becomes a cost‐effective solution.

- Pooling

Custom 5.a: The manufacturer recommends 0.22 fmol per probe per reaction for fixed methylome panel. On the supplier's advice, for our large custom panel, we have reduced successfully to 0.01 fmol per probe per reaction, which greatly reduces the cost of capture, allowing us to process 20 times more samples for the same cost. This improvement is particularly useful for genetic studies involving large numbers of samples.

Custom 5.b: Methyl enhancer reduces off‐target; the volume added (up to 5 μL) depends on methylation and GC content of the custom panel. Optimization may be needed.

- Hybridization

Custom 6.a: Hybridization temperature depends on the size of the custom panel; optimization may be needed.

Custom 6.b: Hybridization time depends on the size of the custom panel (from 30 min to 4 h or even overnight); optimization may be needed.

- Capture

Custom 7.3: Fast Wash Buffer 1 temperature depends on the custom panel; optimization may be needed.

- Amplification

Custom 8.1: Number of cycles depends on the custom panel size; optimization may be needed.

## Conflict of interest

The authors declare no conflict of interest.

## Author contributions

NI developed the protocol, performed the experiment, and wrote the manuscript. SV provided assistance on the platform environment. JS worked with NI to perform the experiment. CD managed the sequencing service on the platform. JD designed the scientific study and secured funding.

## Data Availability

The optimized protocol developed first on eight samples has been deposited to Protocol Exchange open repository (https://doi.org/10.21203/rs.3.pex‐2159/v1). The procedure described focuses on detailing the biological experiments and is related to the scientific article entitled ‘Detection of DNA methylation signatures through the lens of genomic imprinting’ [7]. The raw data produced can be freely and openly accessed on the EMBL‐EBI ENA portal under study accession ID PRJEB58558 (https://www.ebi.ac.uk/ena/browser/home). Quality control of data such as %GC methylation and alignment on genome target regions has been successfully validated.
